# Commentary: Short-term group schema therapy for mixed personality disorders: an introduction to the treatment protocol

**DOI:** 10.3389/fpsyg.2015.00609

**Published:** 2015-05-08

**Authors:** Susan G. Simpson, Sally A. Skewes, Michiel van Vreeswijk, Rachel Samson

**Affiliations:** ^1^School of Psychology, Social Work and Social Policy, University of South AustraliaAdelaide, SA, Australia; ^2^G-kracht Psychomedisch, Centrum BVDelft, Netherlands

**Keywords:** Schema therapy, personality disorder, treatment protocol, pilot study, group therapy

The evidence base for schema therapy (ST) has grown rapidly in recent years, as applied to a wide range of clinical groups (Masley et al., 2011); for ST with groups see Farrell et al. (2009), Simpson et al. (2010), Farrell and Shaw (2012), van Vreeswijk et al. (2012), Renner et al. (2013), Videler et al. (2014). Variations exist between group schema therapy protocols utilized across clinical settings and client groups. It is therefore imperative that researchers describe treatment protocols in detail.

A recent pilot study was published on the outpatient treatment of mixed personality disorders (Skewes et al., 2015), which demonstrated low attrition and clinical improvement over 20 weekly 60-min sessions. In addition, up to 5 × 30-min individual sessions are provided through the duration of the group for those who require additional support, especially during crises. This also includes a session provided at mid-therapy in order to provide feedback on progress to date and to focus goals for the second half of therapy. The purpose of this paper is to describe the treatment protocol utilized in this trial.

The group is led by two therapists who alternate the role of taking the lead in session exercises, with the other focused on maintaining connection between participants, mainly through the use of eye-contact and non-verbal gestures. Therapist couples are able to model connection and confrontation through the way in which they communicate with each other and with group members. Therapists must balance the need to be “genuine” and consistent with their own individual therapeutic “style,” with the need to collaborate as a cohesive “parent couple,” guiding the group according to schema therapy “limited reparenting” principles.

The schema model is applied in a flexible manner that requires group leaders to respond to modes as they appear on a moment-to-moment basis. Whereas participants with BPD often experience frequent mode “flipping,” those with cluster C personality disorders, tend to be overly fused to an avoidant or overcompensatory coping mode (e.g., “Detached Protector”; “Perfectionistic Overcontroller”). Therapists are required to respond to the needs of clients flexibly, and to be guided by sophisticated case conceptualizations that facilitate recognition of the range of modes present in a mixed personality disordered group. In order to ensure treatment fidelity and to check that all four aspects of the schema model (cognitive, behavioral, experiential, and limited reparenting) are covered adequately, sessions are monitored regularly by an accredited group trainer using the Group Schema Therapy Competency Rating Scale (Zarbock et al., 2014). A condition of participation in the group is that contact with external mental health professionals is limited to medical or psychiatric monitoring only.

Mode “check-in points” are used at regular intervals during sessions, which involves a 5 minute mindfulness exercise to facilitate moment-to-moment awareness of modes and associated feeling-states (van Vreeswijk et al., 2014).

A summary of the material incorporated within sessions is summarized in Table 1. Some exercises can be found in Farrell et al. (2014).

**Table 1:** **Summary of schema therapy group protocol for mixed personality disorders**.

Sessions 1–5	Establish group safety and sense of belonging, through group connection and safety-focused imagery exercises whereby group members are encouraged to generate and embellish an imaginary place where they can all find a sense of safety and protection, incorporating individualized elements to fit each person's needs. Psychoeducation regarding childhood needs, schemas and modes and ways of recognizing schema mode triggering. Group exploration of how schemas and modes developed through early family and cultural experiences and link this to current triggers and coping modes. Modes are introduced through a roleplay demonstration, whereby participants are invited to play the part of the different mode “characters.” Roleplay exercises used to develop recognition of modes, with attendant physical and postural signs. Simple schema mode diaries and flashcards introduced to increase awareness of schemas/modes. Introduction to concept of Healthy Adult (HA) and differentiation from coping modes. Exploration of “rational” vs. empathy/compassion-based responses through role-play. Fun activities for the growth of Happy Child.
Sessions 6–10	Interactive discussion regarding the role and signs of Detached Protector mode (DP). One DP experiential group exercise involves two volunteers wearing a motorcycle helmet or mask whilst group discussion continues, to increase awareness of this mode in self and others. Group are encouraged to predict potential in-session triggers for DP and to consider how they can reconnect with group members in DP mode. Pros and Cons exercise with DP from individual and group perspectives. Chair work: Bypassing the DP and connecting with the Vulnerable child mode. Psychodrama exercise whereby group members roleplay the Vulnerable Child mode and say one thing to DP about how it stops them from getting needs met (e.g., “What I really need is…”). Physical movement is used to bypass DP. Physical exercises such as blowing up balloons, arm movement exercises etc. facilitate reconnection with body sensations. Introducing the Parent Modes. Experiential exercise used to develop HA messages to counter the Parent modes, similar to the good parent messages exercise from Farrell and Shaw (2012). Identify transgenerational transmission of Parent modes through genograms. Cognitive schema mode change work. Chair work mode work to fight parent modes and reparent child modes. Group participants draw a group representation of the Punitive Mode on large cardboard then identify and write down the individual Punitive messages on paper stuck to the Punitive mode. In small groups (led by group leaders) they are helped to come up with alternative HA messages to fight back against these messages, then practice saying them loudly to the Punitive mode, whilst tearing up the Punitive messages. Whole group imagery rescripting to meet hitherto unmet needs of group members. Group members visualize their Vulnerable child part and identify feelings and unmet needs. This is followed by a break in imagery where group members identify specific reparenting messages that will directly meet those needs. Group returns to previous image and group leaders read out the compassionate messages for each Vulnerable child (and sends away the Punitive mode if necessary). Behavioral goal setting in group context.
Sessions 11–16	Chair work with parent and coping modes and child modes. Participants draw their own “compassionate HA” mode and explore how compassion looks, feels, sounds etc. compared to the “felt-sense” qualities of the coping modes. Chair work to practice self/other compassion with focus on attunement to the underlying needs. Role-play – empathic confrontation of coping modes and learning ways of directly asking for needs to be met by others. Recognizing signs of Angry Child mode and learning to interpret these as signals of unmet needs. Experiential mode work (e.g., chair work; throwing balls at an effigy of Punitive mode whilst verbalizing anger) to practice using anger to fight parent modes.
Sessions 17–20	Historical role play – recognizing attributions and development of coping modes in childhood. Reparenting group imagery work and coaching participants' HA to meet the needs of child self. Imagery rescripting – therapist acting as good parent. Mode role-plays to practice dealing with modes. Dealing with loss and endings – predicting schema triggering and reparenting the Vulnerable and Angry child modes. Cognitive exercise to prepare plan of action for when the Punitive mode fights back. Maintenance plan to continue to validate Vulnerable Child side and to reparent self.

This protocol forms the basis for ongoing research into short-term treatment of mixed personality disorder population.

## Conflict of interest statement

The authors declare that the research was conducted in the absence of any commercial or financial relationships that could be construed as a potential conflict of interest.
